# Novel RNA Markers in Prostate Cancer: Functional Considerations and Clinical Translation

**DOI:** 10.1155/2014/765207

**Published:** 2014-08-28

**Authors:** Julia M. A. Pickl, Doreen Heckmann, Leonie Ratz, Sabine M. Klauck, Holger Sültmann

**Affiliations:** Working Group Cancer Genome Research, German Cancer Research Center (DKFZ) and German Cancer Consortium (DKTK), National Center for Tumor Diseases (NCT), Im Neuenheimer Feld 460, 69120 Heidelberg, Germany

## Abstract

The availability of ultra-high throughput DNA and RNA sequencing technologies in recent years has led to the identification of numerous novel transcripts, whose functions are unknown as yet. Evidence is accumulating that many of these molecules are deregulated in diseases, including prostate cancer, and potentially represent novel targets for diagnosis and therapy. In particular, functional genomic analysis of microRNA (miRNA) and long noncoding RNA (lncRNA) in cancer is likely to contribute insights into tumor development. Here, we compile recent efforts to catalog differential expression of miRNA and lncRNA in prostate cancer and to understand RNA function in tumor progression. We further highlight technologies for molecular characterization of noncoding RNAs and provide an overview of current activities to exploit them for the diagnosis and therapy of this complex tumor.

## 1. Introduction

Prostate cancer (PCa) is the most frequent tumor in men and a major cause of cancer-related morbidity and mortality. The diagnostic application of the prostate specific antigen (PSA) has led to widespread overdiagnosis and subsequent overtreatment of clinically insignificant tumors. This problem will extensively aggravate in the future: with rising life time expectancy, the number of men diagnosed with PCa will increase since the frequency of PCa is steeply rising in elderly men. Thus, there is a huge demand for novel markers for improved PCa diagnosis, prognosis, and therapy decision.

**Figure 1:**
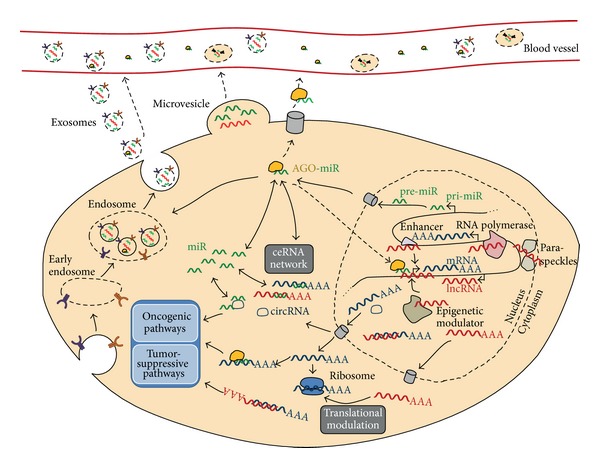
Schematic view of functional networks of different RNA species in cancer. Hypothetical interactions are symbolized with dashed arrows. AGO: Argonaute protein (yellow); miR: miRNA (green); lncRNA: long noncoding RNA (red); mRNA: messenger RNA (blue); circRNA: circular RNA (blue).

Facilitated by the implementation of ultra-high throughput sequencing technologies, the recent discoveries of the ENCODE consortium (http://encodeproject.org/ENCODE/) confirmed that 80% of the human genome is transcribed [1–4]. The GENCODE effort has provided the annotation of more than 9000 lncRNAs [5], as well as micro- (mi-) RNA, small nucleolar (sno) RNA, piwi-interacting (pi) RNA, and others. In parallel, the number of investigations covering the functional analysis of these molecules has increased substantially. Based on these data, it is becoming obvious that many noncoding RNAs are involved in the regulation of gene expression in health and disease, including cancer (Figure 1). For example, global analyses of miRNA expression in cancers revealed robust signatures differentiating between malignant and benign tissues [6], between tumors of several origins, and between primary tumors and metastases [7]. The characterization of miRNA and lncRNA involvement in PCa is likely to result in a better understanding of tumor progression and the identification of novel therapy targets. Here, we provide a summary of recent progress in analyzing noncoding RNA in PCa and in attempting to advance miRNAs and lncRNAs into diagnostic and therapeutic markers.

## 2. miRNA in Prostate Cancer

MiRNAs are ~20–22 nt short noncoding RNAs, which regulate gene expression at the posttranscriptional level by mRNA repression and/or degradation. MiRNAs are transcribed as pri-miRNAs by RNA polymerase II (RNA Pol II) and processed by the RNase III enzyme Drosha to generate precursor miRNAs (pre-miRNAs; Figure 1) [8]. Dicer, another RNase III enzyme, then cleaves the pre-miRNA into ~20–22 long RNA duplexes. Subsequently, the strand with the stable 5′ end is removed, whereas the complementary strand with the less stable 5′ end is incorporated into Argonaute (AGO) proteins to form the RNA induced silencing complex (RISC). MiRNAs guide the RISC to target mRNAs via sequence complementarity [8–11].

Many miRNAs are known to be deregulated in cancer [6]. For instance, miRNAs are differentially expressed between benign prostate and PCa tissues: in a genomewide microarray-based microRNA expression study, 25 miRNAs were found to be deregulated [12]. Here, *miR-375* and *miR-200c* showed highest upregulation in PCa tumor tissue, whereas *miR-221* and *miR-222* were the most downregulated miRNAs [12]. Using a sequencing-based approach, the same group identified 33 miRNAs being up- or downregulated >1.5-fold in PCa tissue [13]. Here, *miR-143* and *miR-145* were the highest downregulated ones in PCa [13]. Consistent with these data, an independent study found *miR-145*, *miR-221*, and *miR-222* among the five most significantly downregulated miRNAs [14], and we identified *miR-375* and *miR-141* as significantly upregulated in PCa tissue [15].

Other analyses revealed the association of miRNA expression with distinct PCa: for instance, *miR-375* expression was reported to be dependent on pT and pN status (TNM staging system) of PCa patients, with the highest enrichment in pN1 tumors [16]. Moreover, ten miRNAs (including *miR-145*, *miR-221*, and *miR-222*) were downregulated and five (including *miR-375*and *miR-96*) upregulated in advanced PCa, whereas *miR-96* was associated with tumor recurrence after radical prostatectomy [17]. MiRNAs are also known to correlate with Gleason score: in high-grade PCa, increased expression of *miR-31*, *miR-182*, and *miR-205* was identified when compared to intermediate-grade PCa [18].

*The functional consequences of differential expression* of various miRNAs have been studied* in vitro* by many groups. Here, we can address only the most prominent examples: *miR-375* was found upregulated in several PCa studies and was known to target Sec23 homolog A(*SEC23A*) [19], which was involved in trafficking from ER to Golgi [20]. The authors suggested that *miR-375*-mediated* SEC23A* downregulation impairs cellular immunogenicity of PCa by reducing* HLA *class I cell surface receptors [19]. *MiR-375* was also shown to downregulate the phosphatase* PHLPP2* and to strongly increase prostate carcinoma cell growth [16]. *MiR-141*, which is upregulated in PCa tissue, was found to downregulate the gene coding for the nuclear receptor subfamily 0, group B, member 2 (*NR0B2*) in prostate epithelial cells. In this way, *miR-141* prevented the inhibitory effects of NR0B2 on androgen receptor- (*AR-*) regulated genes [21]. In contrast, *miR-143* and *miR-145* were found to be downregulated in PCa tissue and to be involved in PCa development via regulation of myosin VI (*MYO6*) [13]. MYO6 was involved in cancer cell dissemination and described as a potential PCa marker [22, 23].

Interestingly, miRNAs, which have not been described as highly deregulated in PCa tissues, were also found to play crucial roles in prostate carcinogenesis. For instance, *miR-200b* attenuated PCa cell growth and invasiveness [24, 25]. Studies in the PCa cell line PC-3 suggested that *miR-200b* mediates the reversal of the epithelial-to-mesenchymal transition (EMT) by targeting the prometastatic transcription factors Zinc finger E-box binding homeobox 1 and 2 (*ZEB1* and* ZEB2*) [25]. Overexpression of *miR-200b* led to increased levels of the epithelial markers E-cadherin, cytokeratins 8 and 18, as well as downregulation of the mesenchymal markers fibronectin and vimentin [25, 26]. Another study found *miR-1* to be reduced with PCa progression. SLUG is a regulator of mesenchymal differentiation and was assumed to repress *miR-1* and *miR-200* transcription whereas *miR-1* and *miR-200* are also able to regulate* SLUG* by a mutually inhibitory feedforward loop [27]. Moreover, *miR-1* was found to act as a mediator of oncogenic pathways in the PCa cell lines PC-3 and LNCaP: the heat-shock protein HSPB1 reduced *miR-1* levels leading to an activation of AR and TGFB1 signaling cascades [28].

MiRNAs are also able to regulate the expression of important* epigenetic marker genes.* For instance, *miR-34b* targeted methyltransferases and deacetylases in PCa [29]. *MiR-34b* is also an example for epigenetic miRNA regulation via a positive feedback loop. *MiR-34b* was found to directly target the AKT pathway like* AKT* itself and its downstream genes* GSK3B*,* CTNNB1*,* MYC*, and* CCND1*, which were involved in cell proliferation and survival [29]. In a PCa xenograft model, *miR-34b* exhibited antitumor properties by decreasing prostate tumor growth [29]. This was probably due to affecting EMT since *miR-34b* expression decreased the mesenchymal markers vimentin, ZO1, N-cadherin, and SNAIL, and increased E-cadherin [29]. Furthermore, *miR-152* was reported to downregulate DNA (cytosine-5)-methyltransferase 1 (*DNMT1*) in LNCaP, PC-3, and MDA-PCa-2b cells.* DNMT1* siRNA caused an increase of *miR-152* expression, suggesting a reciprocal relationship between* DNMT1* and *miR-152*. Increased* DNMT1* and depletion of *miR-152* were assumed to arise during the progression to advanced PCa [30]. The *miR-200* family was downregulated by the histone methyltransferase enhancer of zeste homolog 2 (EZH2) [24]. EZH2 is a component of the polycomb repressive complex 2 (PRC2), which is involved in epigenetic regulation of cell homeostasis.* EZH2* expression was elevated in aggressive forms of PCa [31]. *MiR-200* family on the other hand represses PRC1 proteins BMI1 and RING2. Therefore, increased PRC2 levels lead to an increase of PRC1 expression due to miRNA linkage, suggesting crucial roles for miRNA in regulating the epigenetic silencing machinery [24].

MiRNAs are primarily known as key regulators of active genes. However, it has recently been shown that the expression of the* PTEN *pseudogene (*PTENP1*) is able to rescue the expression levels of its ancestral gene* PTEN* by competing for the same set of miRNAs (*miR-20a*, *miR-19b*, *miR-21*, *miR-26a*, and *miR-214*) [32]. Similarly, the pseudogene* KRASP1* derepressed* KRAS *in DU145 cells, probably by sharing common sites for *miR-143* and* let-7* [32]. Thus, expressed pseudogenes can display tumor suppressive or oncogenic properties by acting as* competitive endogenous RNAs (ceRNAs)* for their corresponding active genes: the increased number of miRNA binding sites upon pseudogene expression was able to titrate the pool of available miRNAs in PCa cells, and miRNA concentrations are regulated by the abundances of their target genes. In this concept, target RNAs are not only passive substrates of miRNAs but also active players in the regulatory networks leading to PCa progression.

The identification of their target genes is essential to estimate the potential of miRNAs as biomarkers. Many* bioinformatic tools* are able to predict miRNA target genes (Table 1). However, since these tools are based on certain assumptions (e.g., base complementarity in the 3′UTR, thermodynamic stability, target-site accessibility, and evolutionary conservation of miRNA binding sites), their predictions are accompanied by large numbers of false positives. Thus, experimental approaches for targetome identification* in vitro* are required. For example, using AGO-RNA immunoprecipitation, followed by sequencing* (AGO-RIP-Seq)* AGO2/miRNA/RNA target complexes are captured from cells stably expressing a miRNA of interest. MiRNA-associated targets are then identified and quantified by deep transcriptome sequencing. AGO-RIP-Seq also enables the identification of novel nonannotated RNA targets like lncRNAs or pseudogenes. Other methods (*AGO-HITS-CLIP* [33, 34],* AGO-PAR-CLIP* [35], and* CLASH* [36]) use crosslinking of the AGO complex to obtain more information about miRNA binding to target genes. These techniques have revealed that some miRNAs can also bind their targets in the coding regions of genes and that other miRNAs do not require base complementarity at all in order to regulate target gene expression [33–36]. These methods are rather novel, and systematic studies reporting the experimental analysis of genomewide miRNA targetomes in PCa have not been described to the best of our knowledge.

MiRNAs are also known to be present in the nucleus [37–40]. Nuclear miRNAs are considered as regulators of gene expression at the transcriptional level (reviewed in [41]). For instance, miRNAs are supposed to bind to promoter sequences in an AGO-dependent manner to regulate gene transcription in the PCa cell line PC-3. This hypothesis was supported by the finding that AGO1-bound promoter sequences contain putative miRNA binding sites [39] and suggest a role for miRNAs in PCa not only in the cytoplasm, but also in the nucleus.

## 3. Long Noncoding RNA in Prostate Cancer

*Long noncoding RNAs (lncRNAs)* are defined by their length, which ranges from 200 bases up to 100 kb. LncRNAs have adopted the transcriptional machinery from protein-coding genes (Figure 1): they are primarily transcribed by RNA Pol II, frequently contain a 5′ terminal methylguanosine cap, and are often spliced and polyadenylated [42]. However, a set of nonpolyadenylated lncRNAs has also been identified to be transcribed from RNA polymerase III promoters [43, 44]. The fact that lncRNAs have been found to bind to ribosomes raises the possibility that they are potentially translatable into proteins. Indeed, ribosome profiling [45] and mass spectrometry approaches [46] detected small peptides potentially derived from lncRNAs. However, the lack of conserved long open reading frames supports the idea that lncRNAs do not encode proteins but rather exert regulatory functions. LncRNAs are cataloged according to their genomic localization: long* intergenic noncoding RNAs (lincRNAs)* are derived from regions that are devoid of other genes, whereas* intronic lncRNAs* are transcribed from introns of protein coding genes. LncRNAs originating from opposite strands of the same genomic locus are called* sense/antisense pairs* and are divided into convergent (head-to-head), divergent (tail-to-tail), and fully overlapping transcripts [47]. LncRNAs are expressed at much lower levels and with much higher cell-type specificity than mRNAs [5]. Although lncRNAs are observed in many species, their degrees of evolutionary conservation are low, and some of the human lncRNAs are thought to have arisen solely in primates [5].

In PCa, the most prominent and clinically relevant RNA biomarker is the antisense lncRNA* PCA3*, which is overexpressed in >95% of primary tumors [48].* PCA3* is a diagnostic biomarker detectable in urine [49], with higher specificity compared to PSA [50]. One of the first lncRNAs suggested as a marker for PCa incidence, androgen-dependent proliferation, and antiapoptotic signaling in cell lines was the PCa gene expression marker 1 (*PCGEM1*) [51–54]. Overexpression of* PCGEM1* in tumor cells inhibited PARP (poly-ADP-ribose-polymerase) cleavage and delayed the induction of p53 and p21 [52], resulting in increased chemoresistance.* PCGEM1* was found to bind to AR at methylated K349 and to regulate the expression of AR target genes, which stimulated tumor growth in a PCa xenograft mouse model [54]. Additionally, two single-nucleotide polymorphisms (SNP) contributing to cancer risk were identified in* PCGEM1* [55].

In a more recent and comprehensive study, Prensner and colleagues, by analyzing the transcriptomes of 102 prostate tumors and cell lines using next generation sequencing [56], identified 121 so far nonannotated PCa-associated noncoding RNA transcripts (PCATs).* PCAT-1* was upregulated in a subset of metastatic and high-grade localized prostate tumors.* PCAT-1*, a transcriptional target of* EZH2*, stimulated cell proliferation [56] and impaired homologous recombination by repressing of the* BRCA2* gene [57]. PCa noncoding RNA 1 (*PRNCR1*;* PCAT-8*), which is located immediately adjacent to* PCAT-1* and the* c-MYC* oncogene at the well-known PCa risk locus on chromosome 8q24 [58, 59], was identified to be upregulated in aggressive prostate tumors [54].* PRNCR1*-bound DOT1-like histone H3K79 methyltransferase (DOT1L) methylated the AR at position K349, which mediated the interaction of AR with* PCGEM1*, thereby resulting in ligand-independent activation of AR signaling and cell proliferation [54].* PCAT-18* was recently suggested as a biomarker for metastatic PCa [60]. Knockdown of *PCAT-18* decreased cell proliferation, migration, and invasion but also triggered apoptosis by promoting caspase activity [60]. In another study,* PCAT-114* (*SCHLAP1*) expression correlated with high-grade, metastatic PCa (see below). By analyzing the transcriptomes of 14 prostate tumors and adjacent benign tissues in another genomewide approach, 137 new lncRNAs were identified [61]. One of these, the metastasis associated in lung adenocarcinoma transcript 1 (*MALAT-1*), was suggested to be a novel marker for PCa diagnosis [62]: high expression of* MALAT-1* correlated with high Gleason score, PSA, tumor stage, and castration-resistant PCa (CRPC).

The majority of PCa are driven by androgen signaling. Various studies have highlighted the functions of androgen-responsive or AR-interacting lncRNAs: Takayama and colleagues identified the antisense lncRNA C-terminal binding protein (*CTBP1-AS*) as highly upregulated in PCa, by repressing its sense gene* CTBP1*, an AR corepressor. This is achieved by recruitment of the transcriptional repressor SFPQ (splicing factor proline/glutamine-rich) and histone deacetylases to the promoter of* CTBP1* [63], thereby promoting androgen-dependent and castration-resistant tumor growth [63]. The PCa-upregulated lncRNA* PlncRNA-1* modulates apoptosis and proliferation through regulation of the AR [64]. Recently,* PCAT-29* was identified as an androgen-repressed suppressor of growth and metastases of prostate tumors [65]. The same group contradicted the prognostic value of* PCGEM1* and* PRNCR1* in aggressive PCa [54]: in a large high-risk PCa patient cohort, Prensner and colleagues could not find any evidence for the binding of these lncRNAs to the AR [66].

As lncRNAs do not encode proteins, their functions are closely associated with their abundance. Bioinformatic tools such as the* “guilt by association analysis”* [67] are capable of predicting the function of an lncRNA. This method uses coexpression analysis of lncRNAs with protein coding genes in signaling pathways to predict the functions of the lncRNAs. This approach has led to the prediction of various roles for lncRNAs, ranging from stem cell pluripotency to cancer [42]. Besides such bioinformatic approaches and databases listing annotated lncRNAs and functional assignment (Table 1), several crosslinking methodologies have been developed to experimentally elucidate the interactomes of lncRNAs* in vitro*. Chromatin isolation with RNA purification* (ChIRP)* [68] and capture analysis of RNA targets* (CHART)* [69] are able to copurify lncRNA-bound proteins and DNA in juxtaposition. In DHT- (dihydrotestosterone-) stimulated LNCaP PCa cells, ChIRP uncovered thousands of PCGEM1 occupancy sites, colocalizing with ARE and H3K4me1-marked enhancers [54].

The regulatory potential of lncRNAs, in particular lincRNAs, has been best characterized in the context of their roles as* epigenetic modulators*. It was observed that 20% of cellular lncRNAs are associated with specific chromatin modification complexes, such as PRC2, which mediates histone H3 lysine 27 trimethylation (H3K27me3) [70, 71].* ANRIL*, an lncRNA overexpressed in PCa, mediated epigenetic silencing of the adjacent tumor suppressor* p15* via H3K27 trimethylation of the* CDKN2A* (*INK4A/ARF*) locus by directly interacting with SUZ12, a component of PRC2, and CBX7, a PRC1-associated chromodomain-containing protein [72, 73]. In aggressive PCa, aberrantly high expression of* PCAT-114* inhibited SWI/SNF-mediated epigenetic regulation, due to direct disruption of SNF5 activity, a core unit of the SWI/SNF complex [74]. As it has been shown that the expression of PCa-associated lncRNAs can be regulated by long terminal repeats (LTRs) [75],* PCAT-114* might be regulated through an LTR in its 5′UTR (own unpublished observations).

Currently, there is little evidence for a direct interaction between lncRNAs and DNA [68]. In contrast, the crosstalk of lncRNA with other RNA species has been studied in detail [35, 76]. Antisense lncRNAs can regulate the stability and translation of complementary mRNAs. For example, translation of the* UCHL1* mRNA was found to be regulated by its antisense lncRNA through complementary 5′ overlap and an embedded inverted* SINEB2* element [77]. In another example, imperfect base pairing between* Alu* elements in lncRNAs and the 3′UTR of translationally active mRNAs resulted in a double-stranded RNA, which is bound by staufen double-stranded RNA binding protein 1 (STAU1) and subsequently targeted for degradation [78].

The ceRNA concept [32] also suggests that lncRNAs—like mRNAs—act as natural sponges (Figure 1). In this context, a long noncoding* circular RNA (circRNA)* was identified to harbor more than 70 target sites for *miR-7*. Binding of this miRNA to the sponge circRNA suppressed miRNA activity, resulting in miRNA target derepression [79, 80]. CircRNA datasets are publically available via http://www.circbase.org/ (Table 1).

The noncoding part of the human genome offers comprehensive prospects to identify the genetic factors and mechanisms, which are critical for PCa initiation, development, and progression. However, a recent study suggests that a particular lncRNA may exert both oncogenic and tumor-suppressive functions in PCa [81]. This indicates that lncRNA action is highly context-dependent, and further efforts are required to understand the increasing complexity of lncRNA function in health and disease.

## 4. PCa Diagnosis and Treatment Monitoring Using Circulating RNA Markers 

The remarkable stability of noncoding RNAs prompted efforts to evaluate their potential as diagnostic or prognostic molecular markers. In particular, genetic material released from tumor cells into the blood or other body fluids (“liquid biopsy”) could provide a rich source of such markers that can be exploited to gain insights into the molecular alterations associated with disease progression [82]. In PCa, liquid biopsies could be highly valuable since the heterogeneity of the tumors often precludes precise staging and grading. In addition, a faithful representation of tumor aggressiveness in easily accessible liquids could lead to an improved therapy decision.

*MiRNAs circulating freely* in blood have been studied as potential biomarkers for PCa [83, 84]. Several miRNAs were demonstrated to discriminate between PCa and healthy controls or patients with benign prostate hyperplasia (BPH) [83, 85], as well as between localized and metastatic PCa [86]. Brase et al. observed an overall higher level of circulating miRNAs in serum samples of patients with advanced PCa compared to those with primary PCa [15]. Of 667 miRNAs screened, 69 miRNAs were found to be more abundant in sera of lymph-node positive versus negative PCa patients. *MiR-375* and *miR-141* showed statistically significant correlation with clinical patient parameters and were identified as the best serum markers for aggressive PCa [15]. Significantly higher abundance of *miR-141* in localized PCa compared to metastatic disease in plasma samples was also seen by other groups [87, 88], while *miR-21* distinguished best between early-stage disease and healthy controls and was correlated with resistance to docetaxel [89]. Serum *miR-194* correlated with biochemical recurrence and poor prognosis [88]. The first clinical studies are ongoing which examine the benefit of circulating miRNAs for PCa diagnosis and disease monitoring (Table 2). These include investigations to determine whether specific miRNA profiles are related to PCa outcome in high-risk PCa patients as well as screening studies for novel markers (including miRNA and lncRNA) associated with tumor progression in general (e.g., http://www.mdanderson.org/) or with locally recurrent or metastatic PCa. Furthermore, the potential of circulating miRNA markers as tools for companion diagnostics in clinical trials (prediction of therapy response against abiraterone or combination therapies; Table 2) is currently being explored.

Two recent studies suggested the detection of transcript levels of selected mRNAs in whole-blood samples of patients with CRPC as useful prognostic markers [90, 91]: a panel consisting of six* circulating mRNA* markers (*ABL2*,* C1QA*,* SEMA4D*,* TIMP1*,* ITGAL*, and* CDKN1A*) predicted survival when compared to clinical and histopathological parameters of prognostic significance in CRPC [91]. Poor prognosis and poor overall survival of patients with CRPC were correlated with a nine-gene model (*RHAG*,* CA1*,* HEPACAM2*,* SNCA*,* HEMGN*,* SOX6*,* TMEM56*,* OSBP2*, and* RHD*) [90]. The determination of* PCA3* in peripheral blood in combination with increased PSA level (>10 ng/mL) showed high specificity and improved diagnostic accuracy to distinguish BPH from PCa [92]. Since PCa often carries genomic rearrangements involving androgen-regulated genes, the role of rearrangement-associated biomarkers could also be exploited for diagnostic purposes. Urine-based combined detection of* PCA3* and* TMPRSS2:ERG* DNA was used for improved risk stratification of men with elevated serum PSA levels to guide further disease management [93], and changes in* PCA3* and* TMPRSS2:ERG* levels are currently used as marker to evaluate the effect of treatment with the gonadotropin-releasing hormone agonist Triptorelin (Decapeptyl) in a Phase III multicenter study in patients with advanced PCa (NCT01020448; https://clinicaltrials.gov/). A multiplex marker panel consisting of* PCA3*,* TMPRSS2:ERG*, annexin A3, sarcosine, and serum PSA showed improved diagnostic accuracy for PCa [94].

*Circulating miRNAs* have been found to be present in* microvesicles* such as* exosomes* (Figure 1). Exosomes are nanosized (40–100 nm) extracellular vesicles secreted from cells via the endosomal pathway. Tumor cells release exosomes at increased levels, which are then detectable in different body fluids. Exosomal miRNAs reflect the miRNA signature of the tumor, and differences in miRNA content and expression were found in exosomes derived from cancerous versus noncancerous cell lines and in tumor-derived exosomes compared to those from healthy individuals [95–97]: in PC-3 cell lines, miRNA profiling identified 31 miRNAs that were exclusively present in exosomes [96]. In larger extracellular vesicles (>200 nm), *miR-141*, *miR-375*, and *miR-205*, known to be associated with PCa progression, and *miR-31* (which targets AR) were differentially expressed [97]. So far, there are only few reports analyzing miRNAs in microvesicles derived from PCa patients. However, a recent study found differential expression of eleven miRNAs, of which *miR-107* exhibited the largest differential expression in circulating microvesicles from PCa patients compared to controls [86]. Additionally, 16 miRNAs were found to be enriched in microvesicles of patients with metastatic PCa compared to patients with organ-confined disease [86]. In this study, *miR-375* and *miR-141* were significantly enriched in plasma-derived microvesicles from patients with metastatic disease. Exosomal* TMPRSS2:ERG* and* PCA3* mRNA from urine samples of untreated PCa patients have also been proposed as a potential diagnostic test [98].

All exosomal content is derived from their parental cells. However, the precise mechanism of miRNA/lncRNA/protein delivery into exosomes is not fully understood. Notably, the RNA and protein cargo within exosomes was shown to be functionally active in recipient cells, which suggests that tumor cells can exploit circulating exosomes as a means to “communicate” with their regional or distal environment [95, 99, 100].

## 5. Preclinical Studies for ncRNA-Based PCa Therapy

With the identification of PCa-specific miRNA expression patterns and the understanding of their role in PCa biology, novel opportunities for miRNA-based therapies arise. These include targeting the 3′UTRs of oncogenes using specific miRNAs, restoring normal intracellular miRNA pools, or enhancing therapeutic response by combining miRNA with existing anticancer drugs, attempting to exploit the concept of synthetic lethality. In this section, we highlight* in vivo* studies that support the implementation of miRNA-based therapeutics (Table 3).

The ability of* let-7* to inhibit tumor initiation was first demonstrated in a murine CD44-positive xenograft model, where overexpression and therapeutic injection of* let-7* resulted in delayed tumor formation [101–103]. Intratumoral administration of *miR-199a-3p* mimics into a murine PCa xenograft model led to decreased expression of the oncogene Aurora kinase A (*AurkA*), which is associated with a malignant PCa phenotype [104]. In another murine xenograft model, using transfected PCa cell lines [103], *miR-128* showed antiproliferative as well as proapoptotic effects and negatively regulated PCa stem cells by directly targeting the stem cell related genes* Bmi-1* and* Nanog* (Nanog homeobox). Additionally, clonogenic and tumorigenic properties of PCa cells were inhibited in a dose-dependent manner by different levels of endogenous *miR-128*. *MiR-16* replacement therapy in a luciferase PCa xenograft substantially reduced bone metastatic tumor growth compared to untreated mice [105]. *MiR-26a*, which is downregulated in PCa, was shown to inhibit growth and metastatic progression of human-tumor xenografts, probably by targeting lin-28 homolog B (*Lin28B*) and zinc finger CCHC domain containing 11* (ZCCHC11)* [106]. Finally, downregulation of the lncRNA* MALAT-1* delayed tumor growth* in vivo* and reduced metastasis of PCa xenografts in castrated male nude mice [62].

Intratumoral and systemic injection of *miR-34a* into mice grafted with human PCa tumors resulted in reduced tumor volumes and lung metastasis and was associated with prolonged survival by suppressing the adhesion molecule CD44 [107]. Deregulated *miR-34a* expression was also linked to drug resistance, showing a sevenfold decrease in paclitaxel-resistant PC-3 cells compared to wild-type PC-3 cells [108]. Overexpression of *miR-34a* in paclitaxel-resistant PC-3 cells recovered paclitaxel sensitivity, while *miR-34a* antagomir transfection attenuated it [108].

Liu et al. [107] and Kojima et al. [108] presented a rationale for *miR-34a-*based therapy to target PCa stem cells and drug-resistant PCa. *MiR-34a* has emerged as therapeutical agent under the name MRX34, a liposome-encapsulated *miR-34* mimic (http://www.mirnatherapeutics.com) and is currently being studied in a multicenter Phase I clinical trial in patients with unresectable primary liver cancer and liver metastases derived from tumors of other primary origin (NCT01829971; https://clinicaltrials.gov/).

Restoration of *miR-30b* expression by src tyrosine kinase (Src) inhibitors was able to prolong survival and reduce metastatic disease of VCaP-derived xenograft mice by negatively regulating* TMPRSS2:ERG* and downregulating EMT-associated as well as ERG target genes [109]. Consequently, inhibitors of the AR activator Src are currently being tested in Phase I and II clinical trials for the treatment of PCa patients. Similar to the reconstitution of silenced miRNAs, suppression of overexpressed miRNAs was also shown to be a potential therapeutic approach: Mercatelli et al. established an antagomir targeting *miR-221* [110]. Inverse correlation of *miR-221* and p27 protein level was validated in primary cells from 18 patients with stage II-III PCa. In mouse xenograft models, intratumoral injection of a *miR-221* antagomir led to significantly reduced tumor growth, which was correlated with p27 upregulation [110].

There are currently no clinical trials studying miRNAs as therapeutics in human PCa. However, the data from these and other preclinical* in vivo* models using miRNA-based therapeutic approaches provide promising results for potential future applications in human PCa.

## 6. Summary

The advent of ultra-high throughput sequencing technologies has caused a vast expansion of the catalog of noncoding RNA molecules. Thus, on the one hand, we are closer to an overview of the transcriptional landscapes in health and disease. On the other hand, the complexity of regulatory factors has grown substantially, and we have just started to uncover some underlying principles: although miRNAs are probably the best investigated class of noncoding RNAs, the recent discoveries of complex miRNA and ceRNA networks and nuclear miRNAs indicate that our understanding of miRNAs in PCa progression is far from complete. Similarly, the analysis of lncRNAs has just commenced, and the roles of the majority of* PCATs* and other lncRNAs in PCa remain to be investigated. The reports on novel diagnostic PCa biomarkers from liquid biopsies are encouraging. However, the data are mostly based on discovery-driven approaches that frequently have limited validity due to small sample sizes, nonstandardized settings, and different experimental designs. Thus, the diagnostic and therapeutic benefits of many potential RNA markers in PCa still need to be proven in well-controlled and standardized large-scale prospective clinical studies.

## Figures and Tables

**Table 1:** Databases and tools for functional annotation of miRNAs and lncRNAs.

Database name	Description	URL
**miRNA**		
TargetScan	miRNA target prediction based on sequence complementarity	http://www.targetscan.org/
PITA	miRNA target prediction based on target site accessibility	http://genie.weizmann.ac.il/pubs/mir07/mir07_data.html
MiRecords	Collection of miRNA target prediction programs	http://mirecords.biolead.org/
MicroPIR	Prediction of miRNA-promoter interactions	http://www4a.biotec.or.th/micropir/
miRvestigator	Identification of miRNAs responsible for coregulated gene expression patterns discovered through transcriptome profiling	http://baliga.systemsbiology.net/drupal/content/mirvestigator-web-application-identify-mirnas-responsible-co-regulated-gene-expression
DIANA miRPath v.2.0	Combinatorial effect of microRNAs in pathways	http://diana.imis.athena-innovation.gr/DianaTools/index.php?r=mirpath/index
miRGator v2.0	Integrated system for functional investigation of microRNAs	http://mirgator.kobic.re.kr/
mirConnX	Condition-specific mRNA-microRNA network integrator	http://mirconnx.csb.pitt.edu/job_config
MAGIA	Tool for miRNA and genes integrated analysis	http://gencomp.bio.unipd.it/magia/start/

**lncRNA**		
ChIPBase	Platform decoding transcription factor binding maps, expression profiles, and transcriptional regulation of ncRNAs from ChIP-Seq data	http://deepbase.sysu.edu.cn/chipbase/
DIANA-LncBase	Database for experimentally verified and computationally predicted miRNA targets on lncRNAs	http://www.microrna.gr/LncBase
lncRNome	Biologically oriented knowledge base for lncRNAs in humans	http://genome.igib.res.in/lncRNome
starBase v2.0	RNA interaction networks based on CLIP-Seq data	http://starbase.sysu.edu.cn/
NONCODE v3.0	Expression and functional lncRNA data obtained from reannotated microarray studies	http://www.noncode.org
lncRNAdb	Database of annotated eukaryotic lncRNAs	http://www.lncrnadb.org
LncRNADisease database	lncRNAs associated with a number of diseases	http://www.cuilab.cn/lncrnadisease
Functional lncRNA Database	Mammalian long non-protein-coding transcripts that have been experimentally shown to be both noncoding and functional	http://www.valadkhanlab.org/database
LNCipedia 2.0	Database for annotated human lncRNA transcript sequences and structures	http://www.lncipedia.org
ncRNA Expression Database (NRED)	Integration tool for annotated expression data of ncRNAs from mouse and human	http://jsm-research.imb.uq.edu.au/nred/cgi-bin/ncrnadb.pl
circBase	Database of circular RNAs	http://www.circbase.org
Linc2GO	Functional annotation of human lincRNA based on the ceRNA hypothesis	http://www.bioinfo.tsinghua.edu.cn/~liuke/Linc2GO/index.html
ceRDB-Competing Endogenous mRNA DataBase/CEFINDER	Web-server for identification of ceRNA for a given mRNA target based on the homology of miRNA binding sites present in the 3′UTR sequence	http://www.oncomir.umn.edu/cefinder/

**Table 2:** MiRNA-based biomarker studies in prostate cancer.

Trial title	Objectives	Study type	Source	Trial number
Micro-RNA Expression Profiles in High Risk Prostate Cancer	Correlation of miRNA expression with prostate cancer outcome	Observational retrospective	Radical prostatectomy	NCT01220427 ^ a^

Blood and Tissue Samples From Patients with Locally Recurrent or Metastatic Prostate Cancer	Sequencing of genomic tumor DNA, genotyping, gene expression, and miRNA/noncoding RNA	Observational prospective	Blood, soft tissue or bone metastases	NCT01050504 ^ a^

MicroRNA in Prostate Cancer Progression: Genetic Variation, Genotype-Phenotype Correlation, and Circulating Biomarker	MiRNAs as predictors of progression	Biomarker screening	Tumor versus normal tissue, plasma	MD Anderson Cancer Center^b^

Abiraterone Acetate in Treating Patients with Metastatic Hormone-Resistant Prostate Cancer	MiRNA expression in tumor metastases as biomarker for sensitivity and resistance; correlation of miRNA with response and progression	Interventional, Phase II clinical trial	Blood (biopsy)	NCT01503229 ^ a^

Bicalutamide and Goserelin or Leuprolide Acetate w/wo Cixutumumab in Patients with Newly Diagnosed Metastatic Prostate Cancer	Correlation of miRNA expression and CTC count	Interventional, Phase II clinical trial	Serum	NCT01120236 ^ a^
				

^a^ http://clinicaltrials.gov.

^b^ http://www.mdanderson.org.

**Table 3:** MiRNA-based therapeutics in prostate cancer preclinical trials.

miRNA	Target	Model used	Delivery method	Phenotypes	Reference
Let-7	Myc, RAS	AR-negative mouse xenograft	Let-7c-lentivirus intratumoral injection	Repressed tumor growth	Nadiminty et al. [102]

miR-199a-3p	Aurora kinase A	DU145 mouse xenograft	Agomir intratumoral injection	Reduced tumor growth	Qu et al. [104]

miR-16	CDK1, CDK2	Murine bone metastatic prostate cancer model	Intravenous injections	Reduced tumor development in bone tissue	Takeshita et al. [105]

miR-34a	CD44, SIRT1	CD44^+^ mouse xenograft	Intratumoral and systemic injection of miR-34a oligo	Reduced tumor volume and lung metastasis, prolonged survival	Liu et al. [107]

miR-221	p27	PC-3 mouse xenograft	Intratumoral injections of miR-221/miR-222 antagomirs	Reduced tumor growth	Mercatelli et al. [110]

## Abbreviations

BPH: Benign prostate hyperplasia
ceRNA: Competitive endogenous RNA
CHART: Capture analysis of RNA targets
ChIP: Chromatin immunoprecipitation
ChIRP: Chromatin isolation with RNA purification
circRNA: Circular RNA
CLASH: Crosslinking, ligation, and sequencing of hybrids
CRPC: Castration-resistant prostate cancer
DHT: Dihydrotestosterone
EMT: Epithelial-to-mesenchymal transition
HITS-CLIP: High throughput sequencing of RNA isolated by UV crosslinking immunoprecipitation
lincRNAs: Long intergenic noncoding RNAs
lncRNA: Long noncoding RNA
miRNA: microRNAs
PAR-CLIP: Photoactivatable-ribonucleoside-enhanced crosslinking and immunoprecipitation
PCa: Prostate cancer
PCAT: Prostate cancer associated transcript (nonprotein coding)
piRNA: piwi-interacting RNA
PRC2: Polycomb repressive complex 2
PSA: Prostate-specific antigen
RIP-Seq: RNA immunoprecipitation sequencing
RISC: RNA-induced silencing complex.
